# Association of sleep duration and risk of mental disorder: a systematic review and meta-analysis

**DOI:** 10.1007/s11325-023-02905-1

**Published:** 2023-08-29

**Authors:** Jinhe Zhang, Mengyang He, Xuan Wang, Hairong Jiang, Jinchang Huang, Sixiang Liang

**Affiliations:** 1 The National Clinical Research Center for Mental Disorders & Beijing Key Laboratory of Mental Disorders, Beijing Anding Hospital, Capital Medical University & the Advanced Innovation Center for Human Brain Protection, Capital Medical University, Beijing, China; 2 Graduate School, Beijing University of Chinese Medicine, Beijing, China; 3 Beijing University of Chinese Medicine Third Affiliated Hospital, Beijing, China

**Keywords:** Sleep duration, Depression, Meta-analysis, Mental disorders, Adults

## Abstract

**Background:**

The effects of sleep duration on the development of mental illness remain controversial. Therefore, it is necessary to identify the effects of long or short sleep duration on psychological disorders, which could reveal new ways for preventing and treating mental health conditions cheaply.

**Methods:**

Identifying published papers was accomplished by using the following five English databases on March 16, 2022: PubMed, MEDLINE, Embase, Web of Science databases, and Scopus. Cross-sectional and cohort studies were considered if they evaluated the association of sleep duration with all kinds of mental illness in adults. We excluded case reports, editorials, narrative reviews, and studies without detailed information on sleep duration. Summary effect-size estimates were expressed as risk ratios (RRs) or odds ratios (ORs) with 95% confidence intervals and were evaluated using random-effect models. Mantel-Haenszel’s random-effects model was used to estimate the inconsistency index (*I*^2^) and Tau^2^ index (measurement of heterogeneity).

**Results:**

A total of 52 studies were included in this analysis, consisting of 14 cohort studies and 38 cross-sectional studies. These studies involved a combined sample size of 1,407,891 participants who met the inclusion criteria. Cohort (adjusted RR = 1.42, 95% CI: 1.26–1.60, *P* < .001, *I*^2^ = 37.6%, Tau^2^ = 0.014) and cross-sectional studies (adjusted OR = 1.67, 95% CI: 1.57–1.77, *P* < .001, *I*^2^ = 79.7%, Tau^2^ = 0.060) concluded that short sleep duration increased mental disorder risks. The same conclusions were acquired in the subgroup analysis, especially for depression (adjusted RR = 1.43, 95% CI: 1.24–1.65, *P* < .001, *I*^2^ = 80.4%, Tau^2^ = 0.082), anxiety (adjusted RR = 1.30, 95% CI: 1.04–1.63, *P* = .002, *I*^2^ = 0.0%, Tau^2^ = 0.000), and PTSD (adjusted RR = 1.35, 95% CI: 1.04–1.76, *P* = .022, *I*^2^ = 24.1%, Tau^2^ = 0.013) in cohort studies. The results of subgroup analysis indicated that long sleep duration was not a risk factor for depression (adjusted RR = 1.15, 95% CI: 0.98–1.34, *P* = .088, *I*^2^ = 63.4%, Tau^2^ = 0.045) and anxiety (adjusted RR = 1.37, 95% CI: 0.93–2.03, *P* = .114, *I*^2^ = 0.0%, Tau^2^ = 0.000).

**Conclusions:**

Short sleep duration, not long sleep duration, is an independent predictor of developing mental disorders, particularly anxiety and depression.

**Supplementary Information:**

The online version contains supplementary material available at 10.1007/s11325-023-02905-1.

## Introduction

The increasing prevalence of mental health disorders is a global issue. In 2019, these disorders accounted for 125 million disability-adjusted life-years [1]. Mental illness affects a significant portion of the global population, with approximately one-eighth suffering from such disorders. Additionally, individuals in post-conflict settings experience mental health problems at a rate of about one in five [2]. The economic effect of mental illness is substantial, including productivity loss and other indirect social expenses that often surpass healthcare expenditures [3]. The World Health Organization estimates that losses from depression and anxiety, the two most common mental health conditions, are upward of $1 trillion annually [2].

In light of growing concerns about mental health, it is crucial that we have a thorough understanding of this topic. According to the World Health Organization (WHO), mental health refers to an individual’s well-being and how they handle stress, reach their potential, learn, and contribute to society. Mental health is a vital aspect of overall well-being as it affects our ability to make decisions, form relationships, and shape the world around us [4]. It also affects communication, functioning, coping mechanisms, and personal development. Recognizing mental health as a basic human right essential for personal growth, community welfare, and socio-economic progress has become increasingly important in recent years. This recognition is evident through its inclusion in sustainable development goals aimed at achieving global development objectives [2].

The prevalence of different mental disorders varies according to gender and age, with anxiety disorders and depression being the most common in both men and women. Depression is a common mental illness around the world, affects people’s health, is linked to conditions like cardiovascular disease and diabetes, and causes significant mortality in the elderly [5–8]. Therefore, identifying potential risk factors for mental disease and intervening to modify long-term exposure to risks for mental health are critical to preventing the development of mental diseases that have serious economic and social consequences.

Most investigations have focused on potential risk factors for mental health related to the residential environment, culture, and lifestyle, such as physical activity, unhealthy diet, alcohol, and drug consumption [9–11]. It has been shown that these factors can affect mental health in various settings. Individuals with mental illness often experience sleep disorders, and genetic analyses have revealed significant genetic correlations between these traits. The study by O’Connell et al. [12] provides evidence that there is substantial polygenic overlap between psychiatric disorders and sleep-associated phenotypes that transcends genetic correlations. Li et al. [13] conducted a longitudinal study using data from the UK Biobank, focusing on participants of European ancestry aged 38–73 years. The results of this study [13] suggest possible genetic mechanisms and structural changes in the brain that may underlie the nonlinear relationship between sleep duration and cognitive and mental health.

As witnesses of the rapid evolution of human society, technological advances, global industrialization and urbanization, and modern lifestyles, including the adoption of unhealthy sleep habits, have led to an increase in the incidence of non-communicable chronic diseases such as mental disorders [9, 14]. Researchers have explored the relationship between sleep duration and psychological illness [15–20]. Sleep maintains human body function and homeostasis by preserving consciousness and cognitive function, sustaining biological rhythm, repairing defense function, and relieving stress [17, 21]. Short sleep duration (SSD) is a risk factor for mental disorders such as depression. A cross-sectional study [15] of 49,317 Chinese older adults suggests that SSD is associated with depressive symptoms in Chinese older adults. Dong et al.’s study [16], which includes adults who participated in the National Health and Nutrition Examination Survey (NHANES) from 2009 to 2016, shows that SSD is independently associated with higher incidence of depression. Findings [22] based on multiethnic populations found that SSD (< 6 h compared to 7–8 h) is independently associated with any psychiatric disorder. However, the effects of long sleep duration (LSD) on the development of mental illness remain controversial. Jing et al. [23] showed that LSD reduces the incidence of depression. In contrast, Plante et al. [24] showed that LSD increases odds of depression. However, several studies [22, 25, 26] concluded that mental disorders, such as depression, anxiety, bipolar disorder (BD), or obsessive-compulsive disorder (OCD), were not associated with LSD.

Based on these contradictory findings, it is necessary to identify the effects of long or short sleep duration on psychological disorders, which may reveal new ways to prevent and treat mental health conditions. Therefore, a meta-analysis was conducted to quantify the relationship between sleep duration and psychological well-being.

## Methods

### Registration and reporting format

The findings were analyzed in accordance with the PRISMA (Preferred Reporting Items for Systematic Reviews and Meta-analyses) guidelines [27] and meta-analysis of MOOSE (Observational Studies in Epidemiology) statement [28] (eTable 1 and eTable 2). Preregistration of the protocol in the PROSPERO database was completed (CRD42022332858).

### Search strategy

Searches were performed in March 2023 based on PubMed, MEDLINE, Embase, Web of Science, and Scopus databases. The PICOS tool was used to guide the search strategy: (P) population: participants with specific sleep duration; (I) intervention: short or long sleep duration; (C) comparator: normal sleep duration; (O) outcomes: all kinds of mental disorders; and (S) study type: cross-sectional and cohort studies. A description of the search strategy is shown in eTable 3. An independent third author (H. J.) verified the accuracy of all searches.

### Selection criteria and study selection

Cross-sectional and cohort studies were considered if they evaluated the association of sleep duration with mental illness in adults. Among the exclusion criteria were case reports, editorials, narrative reviews, and studies that did not involve detailed sleep duration information. We used Endnote 20 literature management software to screen articles that ultimately met the inclusion criteria. The specific selection process contained three steps according to the title, title and abstract, and the final qualified literatures are gradually browsed as the figure.

### Data extraction

Two authors (J. Z. and M. H.) independently extracted the following baseline data from each qualified article, including the first author, year of publication, country where the study was performed, gender, sample size, study type, follow-up years, the age of study subjects, type of mental disorder, career, ascertainment of sleep duration, ascertainment of mental disorders, and other confounding risk factors. We resolved the divergence by re-evaluating original articles together and by involving a third author (J. H.).

### Risk of bias of individual studies

We used the Agency for Healthcare Research and Quality (AHRQ) [29] assessment tool to asses bias in the eligible cross-sectional studies and the Newcastle-Ottawa Scale (NOS) [30] to evaluate cohort studies. Whether the answer to the AHRQ item was “no” or “unclear” would be scored “0,” while “yes” would be scored “1.” A three-grade quality assessment was conducted on the articles: low quality (0–3), moderate quality (4–7), and high quality (8–11). In order to reach a final agreement, differences in the quality of the articles were discussed.

The NOS evaluates cohort studies through three blocks of eight-item methods, specifically including the selection of study population, comparability, exposure evaluation, or outcome evaluation. NOS adopts the semi-quantitative principle of the star system to evaluate the quality of literature, which is fully divided into 9 stars.

### Statistical analyses

The data processing was performed using STATA software version 14.1 for Windows (Stata Corp, College Station, TX, USA). Risk ratios (RRs) or hazard ratios (HRs) were calculated with 95% confidence intervals (CIs) in cohort studies; whereas, odds ratios (ORs) were calculated with 95% CIs in cross-sectional studies to estimate the effect size. We use the formula RR = (1−expHR*ln (1−*r*))/*r* to transform the HRs into RRs and the random-effects model to pool the effect-size estimates. In order to better compare the difference between the two statistics, the *Z*-test proposed by Altman and Bland [31] was performed.

The inconsistency index (I^2^) and another index, *τ*^2^ (Tau^2^), by virtue of the random-effects Mantel-Haenszel model, were both applied to appraise the heterogeneity between studies. When *I*^2^ was greater than 50%, it is considered that there was a significant heterogeneity between studies.

A sequence of subgroup analyses was conducted to make clear the potential sources of between-study heterogeneity. These subgroup analyses constituted various aspects, such as type of mental disorders, study design, age, gender, the level of economic development of the countries, career, ascertainment of sleep duration, level of AHRQ score, and follow-up interval.

To determine the likelihood of publication bias, we also applied Begg’s funnel plot and Egger’s regression asymmetry test. The aim of the scissor’s method is to identify and correct the funnel plot asymmetry caused by publication bias. Based on the hypothesis that publication bias can cause asymmetry of funnel plot, the clipping method uses an iterative method to estimate the number of missing studies, which does not mean estimating the specific number of missing studies but lies in the robustness of the judgment results. After adding some studies, meta-analysis was performed again. If the pooled effect size estimate did not change significantly from that before clipping, it indicated that publication bias had little effect, and the results were relatively robust.

## Results

### Eligible studies

**Fig. 1:**
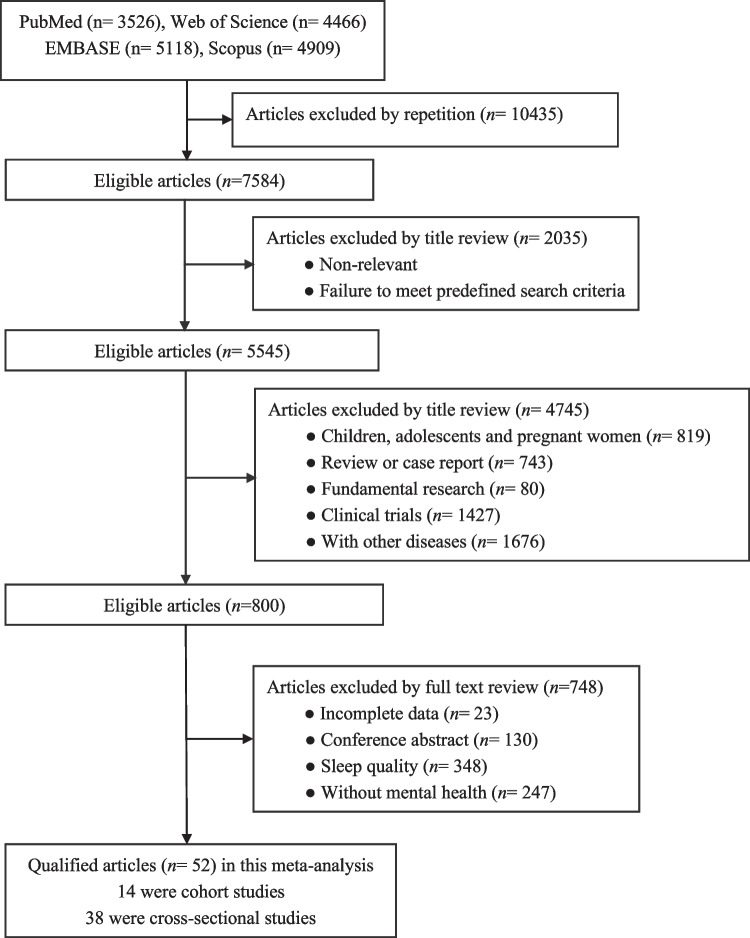
Flow chart of records retrieved, screened, and included in this meta-analysis

We searched 18,091 articles after retrieving the common databases mentioned above using pre-negotiated keywords for sleep duration and mental illness, and 52 studies (14 cohort studies and 38 cross-sectional studies), including 1,407,891 participants satisfied the criteria for inclusion. Figure 1 depicts the comprehensive selection procedure.

### Study characteristics

Table 1 shows the baseline characteristics of the 52 articles [15–20, 22–26, 32–72] included in this meta-analysis. There are fourteen [23, 25, 26, 35, 37, 39, 43, 49, 51, 55, 58, 65, 69, 72] articles belong to cohort studies (three [26, 38, 72] of which also contained data from cross-sectional study), and the number of articles belonging to cross-sectional studies is 38 [15–20, 22, 24, 32–34, 36, 39–42, 44–48, 50, 52–54, 56, 57, 59–64, 66–68, 70, 71] in eligible articles. Of the eligible articles included in this study, a total of 34 articles [16, 18, 20, 22, 24–26, 33–35, 37–40, 43, 45, 47, 51, 54–56, 58–61, 63–70, 72] are attributed to developed countries, and the remaining 18 articles [15, 17, 19, 23, 32, 36, 41, 42, 44, 46, 48–50, 52, 53, 57, 62, 71] are affiliated with developing countries. Among the qualified articles, anxiety was the consequence in 2 articles [25, 66], PTSD was the conclusion in 1 article [64], suicide attempt (SA) was the outcome index and only 1 article [33], suicidal ideation (SI) was the conclusion in 2 articles [18, 60], and there were 42 articles [15–17, 19, 20, 22–24, 26, 32, 34–59, 61–63, 65, 67–72] with depression. Different types of occupations other than the general population were included in the included articles. These occupational types include military personnel [19, 25, 64, 66], college students [35, 41, 48], health care workers [42, 70], and worker groups [59]. The elderly population was mentioned in 14 articles [15, 17, 23, 26, 35, 38, 43, 46, 51, 55, 56, 58, 67, 72]; the middle-aged population in 2 articles [49, 52], and 3 articles [19, 41, 48] involved the young population. Polysomnography (PSG), the objective method, was used to measure sleep duration in 6 articles [37, 51, 55, 56, 58, 65]. And sleep duration was obtained from subjective questionnaire scales (Pittsburgh Sleep Quality Index or Epworth Sleepiness Scale) in the remaining articles. There were 3 qualifying literature articles [24, 48, 58] that dealt only with LSD and 13 articles [19, 25, 32, 33, 35, 37, 41, 42, 45, 50, 61, 65, 66] that dealt only with SSD. SSD was ≤ 5 h in 15 articles [18, 33, 42, 43, 45, 46, 51, 54–56, 60, 64, 65, 67, 70], ≤ 6 h in 28 articles [17, 19, 20, 22, 23, 25, 26, 37, 39, 43–45, 47, 49, 50, 52–54, 59–64, 66, 69, 70, 72], and ≤ 7 h in 20 articles [15, 16, 23, 32, 34–36, 38, 39, 41–43, 49, 52, 57, 64, 65, 68, 71, 72]. There were 5 articles [24, 43, 44, 60, 69] with the LSD of ≥ 10 h, 27 articles [16, 18, 20, 22, 24, 26, 34, 36–40, 44, 47, 49, 52–54, 57, 58, 60, 62, 64, 67, 68, 71, 72] with sleep duration ≥ 9 h, and 19 articles [15, 17, 23, 25, 39, 44, 46, 48, 49, 51, 52, 54–56, 59, 60, 63, 70, 72] with sleep duration ≥ 8 h.

**Table 1:** Main characteristics of the studies conducted on sleep duration and mental disorder risk included in the meta-analysis

Year	First author	Career	Country	Study type	Age (years)	Gender	Sample size	Men	Women	Follow-up years	Mental	Method of sleep duration	Method of mental disorders	Sleep duration	Ref	Adjusted
1997	Chang	Medical student	USA	Cohort	62.6	Male	1053	1053	0	34	Depression	Habit survey questionnaire	Physician reviewers	≤7	> 7	Age at graduation, class year, parental history of depression, measures of temperament, and coffee drinking (cups per day) in Cox proportional hazards analyses
2002	Hidalgo	Medical student	Brazil	Cross-sectional	18–35	Both	342	199	143	0	Mental disorder	ESS	SRQ	< 7	> 7	_
2005	John	General	German	Cross-sectional	18–64	Both	4075	2000	1968	0	Depression	Questionnaires	CIDI	< 5	7–8	Sex, age, and years of school education, with forward stepwise selection of variables. Excluded by the analysis were somatoform disorders
2008	Paudel	General	USA	Cross-sectional	≥ 67	Male	351	351	_	0	Depression	Actigraphy	GDS	≤ 5	6–8	Age, site, race, body mass index, living status, alcohol intake, smoking status, cognitive impairment, physical activity medical conditions, education, instrumental activity of daily living impairment self-reported health status, antidepressant use, benzodiazepine use, and nonbenzodiazepine anxiolytic or hypnotic use
2010	Szklo-Coxe	General	USA	Cohort	33–71	Both	555	333	222	4	Depression	Polysomno-graphically assessed	Zung Self-Rating Depression Scale	< 5.57	≥ 6.82	Age, sex, chronic health conditions, alcohol consumption, cigarette smoking, use of hypnotic agents, caffeine consumption, and body mass index
2010	Yokoyama	General	Japan	Cross-sectional	≥ 65	Both	4997	_	_	4	Depression	Self-reported response to the question	CES-D	< 6	7–8	_
2010	Park	General	Korean	Cross-sectional	18–64	Both	6510	3280	3230	0	MDD	Questionnaires	K-CIDI	5	7	Age, gender, residential area, marital status, education, and employment status, physical activity level, current alcohol use, physical illness, pain /discomfort level, and body mass index
2010	Wada	Physician	Japan	Cross-sectional	> 24	Male	3862	3025	837	0	Depression	Questionnaires	QIDS-SR	< 5	6–7	_
2011	Blasco Fontecilla	General	Spain	Cross-sectional	> 18	Female	1026	484	542	0	SA	Self-assessment	BMLS	≤ 5	7	Gender, age, current MDE, GAD, alcohol use disorders, and the different clusters of PDs
2011	Chang	General	USA	Cross-sectional	51.4 ± 15.8	Both	1204	_	_	0	Depression	Questionnaires	PHQ-2	< 7	7–8	Age, gender, race, education, employment status, income, BMI, history of chronic disease cancer, any exercise in the last month, and current smoking status
2013	Paudel	General	USA	Cohort	≥ 67	Male	2510	2510	_	3	Depression	Actigraphy	GDS	≤ 5	6–8	Age, clinic site, baseline GDS score, health status, education, use of benzodiazepines, and use of antidepressants (in analyses including baseline antidepressant users)
2013	Gehrman	Military personnel	USA	Cohort	33.1 ± 8.3	Both	15204	7519	1524	5	Anxiety	Self-reported	PHQ	< 6	7	Birth year, sex, race/ethnicity, education, marital status, service branch, service component occupation, pay grade general health, BMI, life stressors, smoking status, and problem drinking/CAGE
2013	Sakamoto	Worker	Japan	Cross-sectional	45 ± 11	Both	1197	252	57	0	Depression	Questionnaires	CES-D	< 6	6–7	Age (year, continuous), sex, marital status (married or other), employment type (regular or other) job type (managerial and clerical or technical work), job position (low or middle and high), overtime work (< 10, 10 to < 30 or 230 h/month), one-way commuting time (< 30, 30 to < 60 or 260 min), alcohol consumption (nondrinker, occasional drinker, drinker with a consumption of < 23 or 223 g of ethanol/day), smoking status (nonsmoker, former smoker, or current smoker), leisure-time physical activity (< 120 or 2120 min/week), history of serious diseases including cancer, ischemic heart disease or cerebrovascular disease (yes or no), and history of common diseases including hypertension, diabetes, or dyslipidemia (yes or no)
2013	Swinkels	Veteran	USA	Cross-sectional	37.4 ± 10.0	Both	1640	1307	333	0	PTSD	PSQI	DSM	≤ 5	7–8	Age, minority status, gender, combat exposure, military rank, and number of military tours, in addition health risk behaviors
2014	Maglione	General	USA	Cohort	≥ 70	Female	952	0	952	5	Depression	Actigraphy	GDS	< 5	5–8	_
2014	Taylor	Marine	USA	Cross-sectional	> 18	Both	3175	2562	546	0	GAD	Questionnaires	PHQ	≤ 6	> 6	_
2014	Van Mill	General	Netherland	Cohort	42.7 ± 12.3	Both	1069	356	713	2	Depression	Questionnaires	DSM-IV	≤ 6	7–9	Age, gender, education, alcohol intake, body mass index, number of chronic medical disorders, antidepressants, benzodiazepines, and severity of symptoms
2015	Fernandez	General	USA	Cohort	≥ 20	Both	1137	_	_	7.5	Depression	PSG	Physician diagnosis or treatment of depression	< 6	7	Gender, race, age, body mass index (BMI), obstructive sleep apnea (OSA), hypertension diabetes, caffeine, tobacco-alcohol consumption, and alcohol use disorder, as well as drug use disorder, suicide thoughts or attempts, and feelings of loneliness
2015	Furihata	General	Japan	Cross-sectional	≥ 20	Both	2532	1151	1381	0	Depression	PSQI	CES-D	< 6	7–8	_
2015	Grossi	General	Swedish	Cross-sectional	42 ± 9	Both	420	96	324	0	Depression	KSQ	HADS	≥ 9	< 9	Quality of sleep and other variables that differed between groups, i.e., gender, sick leave (dichotomized as yes vs. no), and use of antidepressants
2015	Lee	General	Korean	Cross-sectional	≥ 19	Male	17,638	7482	10,156	0	Depression	Questionnaires	Questionnaires	≤ 6	7–8	_
2016	Plante	General	USA	Cross-sectional	33–82	Both	3324	1801	1523	0	Depression	Questionnaires	Zung Self-Rating Depression Scale	≥ 9	< 9	Age, sex, body mass index, smoking status, alcohol use, caffeine use, chronic conditions insomnia, sedative drugs, and sleep disordered breathing
2017	Furihata	General	USA	Cross-sectional	≥ 70	Female	6485	_	_	0	Depression	Questionnaires	GDS	< 7	7–9	_
2017	Jackowska	General	UK	Cohort	≥ 50	Both	4545	2063	2482	6	Depression	Questionnaires	CES-D	≤ 5	7–8	Age, sex, relationship status, wealth, presence of limiting long-standing illness, BMI, smoking, alcohol consumption, physical activity, depressive symptoms at baseline, and depression treatment
2017	Li	General	China	Cohort	45–65	Both	7156	_	_	2	Depression	Questionnaires	CESD-10	< 6	7–9	_
2017	Lippman	General	USA	Cross-sectional	> 65	Both	1110	687	423	0	Depression	Questionnaires	CES-D	< 6	6–8	_
2017	Mohan	General	China	Cross-sectional	35–65	Both	9582	4356	5226	0	Depression	Questionnaires	PHQ-9	≤6	7–8	_
2017	Plante	General	USA	Cohort	59 ± 9	Both	891	_	_	4	Depression	PSG	Zung Self-Rating Depression Scale	≥ 9	< 9	Age, sex, body mass index, smoking status, alcohol use, caffeine use, chronic medical conditions, insomnia, sedative hypnotic use, and sleep disordered breathing
2017	Supartini	General	Korean	Cross-sectional	20–69	Male	600	306	294	0	Depression	PSQI	CESD	< 6	6–8	Age, fish consumption, and exercise, socio-demographic and health behavior variables
2017	Thomas	General	USA	Cross-sectional	≥ 65	Female	12,776	_	12,776	0	Mental disorder	BRFSS	BRFSS	< 5	6–8	General health, activity level, weight status, activity limitations, and chronic health conditions, alcohol use, tobacco use, education level, employment status, income level, marital status, ethnicity/race, and age
2017	Wang	General	China	Cross-sectional	19–59	Both	17,320	8420	8900	0	Mental disorder	Questionnaires	GHQ-12	< 7	7–9	Socio-demographics, lifestyle factors, mental health, and multimorbidity
2018	Liu	General	China	Cross-sectional	51.0 ± 10.5	Female	512,891	210,259	302,632	0	Depression	Questionnaires	CIDI	≤ 6	7–8	Residency, age, family mental disorder history, blood pressure, education, income occupation, BMI, marital status, smoking, alcohol, MET statuses, sleep snoring, taking medicine for sleep, daytime, dysfunction, difficulty falling asleep and interrupted sleep, total sleep duration, and disease statuses
2018	Peltzer	General	South Africa	Cross-sectional	≥ 40	Both	4725	2212	2513	0	Depression	Questionnaires	CES-D	< 7	7–8	Age, sex, education, wealth status, tobacco use, alcohol dependence, physical inactivity, inadequate fruit and vegetable consumption, BMI body weight, depression, and PTSD symptoms
2018	Sullivan	General	USA	Cross-sectional	47.5 ± 0.2	Male	20,851	10,216	10,365	0	Depression	Questionnaires	Questionnaires	6	7–9	Age, race, education, marital status, BMI, education, employment, and income
2018	Sun	General	China	Cross-sectional	30–79	Both	512,891	210,285	302,606	0	Depression	Questionnaires	CIDI-SF	≤ 6	7–9	Age, gender, survey sites, marital status, level of education, occupation, living alone and household income per year, alcohol consumption, smoking status, tea consumption, and physical activity; intake frequencies of red meat, fresh fruits vegetables, numbers of chronic disease, body mass index, anxiety, stressful life events, and self-rated health
2019	Ibrahim	Nurse	Saudi Arabia	Cross-sectional	32 ± 7	Both	977	_	_	0	Depression	Questionnaires	DASS-21	≤ 5	≥ 8	_
2019	Ouyang	General	China	Cross-sectional	≥ 45	Both	9529	3183	6346	0	Depression	Questionnaires	CES-D	≤ 6	7–9	_
2020	AI-Ajlouni	General	Jordan	Cross-sectional	18–65	Both	1240	656	583	0	Depression	PSQI	Depression Scale	≤ 7	> 7	Age, gender, region, employment, and physical activity
2020	Chen	General	China	Cross-sectional	18–65	Both	13,678	6159	7609	0	Depression	Questionnaires	PHQ-9	< 7	7–9	_
2020	Jiang	General	China	Cross-sectional	18–79	Male	28,202	11,236	16,966	0	Depression	PSQI	PHQ-2	< 6	7	_
2020	Jing	General	China	Cohort	≥ 60	Both	22,847	11,606	11,241	5	Depression	Questionnaires	CES-D	< 6	7–8	Age, gender, marital status, education, residency, health status, chronic disease status, BMI, smoking, and drinking status
2020	Lai	General	China	Cross-sectional	≥ 65	Both	2620	1076	1544	0	Depression	AIS	HADS	≤ 5	6–7	Age, sex, BMI, education level, living status, cigarette use, alcohol consumption, medical history, and exercise frequency
2020	Li	Students	China	Cross-sectional	16–27	Both	9515	4554	3114	0	Depression	Questionnaires	SDS	7–8	< 7	_
2020	Matsui	General	Japan	Cross-sectional	20–69	Both	8698	_	_	0	Depression	Epworth Sleepiness Scale	CES-D	< 6	7	_
2020	Seow	General	Singapore	Cross-sectional	≥ 18	Both	6126	3068	3058	0	Mental disorder	PSQI	WHM-CIDI	≤ 6	7–8	Sociode mographic/lifestyle factors and sleep quality
2020	Simmons	General	USA	Cross-sectional	48 ± 19	Both	4773	2291	2482	0	SI	Questionnaires	PHQ-9	≤ 4	7	Age, gender, race, education, poverty-to-income ratio, marital status, smoking status, alcohol consumption, and binge\drinking
2020	Tonon	Military personnel	Brazil	Cross-sectional	18.0	Male	236	236	0	0	Depression	PSQI	BDI	< 6	> 6	_
2020	Tubbs	General	USA	Cross-sectional	22–60	Both	1007	388	619	0	Depression	Questionnaires	PHQ-9	< 7	7–8	_
2021	Ko	General	Korean	Cross-sectional	≥ 19	Both	33,481	14,401	19,080	0	SI	Questionnaires	Questionnaires	≤ 5	5–9	_
2022	Ding	General	China	Cross-sectional	≥ 60	Female	1429	0	1429	0	Depression	Questionnaires	Zung Self-Rating Depression Scale	< 6	6–8	Age, BMI, educational level, former occupation, household income, living condition, smoking and drinking habits, hypertension, diabetes, and physical activity
2022	Dong	General	USA	Cross-sectional	≥ 18	Both	25,926	12,764	13,162	0	Depression	Questionnaires	PHQ-9	< 7	7–9	_
2022	Luo	General	China	Cross-sectional	≥ 60	Both	49,317	30,739	18,578	0	Depression	Questionnaires	PHQ-9	< 7	7–8	_

*USA* the United States of America, *UK* the United Kingdom, *CES-D* Center for Epidemiological Studies Depression, *GDS* Geriatric Depression Scale, *PHQ-9* Patient Health Questionnaire, *PCL-C* PTSD checklist, civilian version, *SRQ* self-reporting questionnaire, *ESS* Epworth Sleepiness Scale, *CIDI* World Health Organization Composite International, *K-CIDI* the Korean version of the Composite International Diagnostic Interview, *GAD* generalized anxiety disorder, *QIDS-SR* Quick Inventory Depressive Scale-Self Reported, *BMLS* Beck’s Medical Lethality Scale, *ICD-9* International Classification of Diseases, ninth revision, *BSSI* Beck Scale for Suicide Ideation, *KSQ* Karolinska Sleep Questionnaire, *HADS* Hospital Anxiety and Depression Scale, *BRFSS* Behavioral Risk Factor Surveillance System, *GHQ* General Health Questionnaire, *HADS* Hospital Anxiety and Depression Scale, *DASS-21* Depression Anxiety Stress Scale 21, *AIS* Athens Insomnia Scale, *SDS* Self-Rating Depression Scale, *WHM-CIDI* World Mental Health Composite International Diagnostic, *BDI* Beck Depression Inventory, *SI* suicidal ideation, *PD* panic disorder, *MDD* major depressive disorder, *SA* suicide attempt, *PTSD* post-traumatic stress disorder, *BD* bipolar disorder, *GAD* generalized anxiety disorder

### Results of NOS and AHRQ assessment

The quality of all eligible articles is displayed in eTable 4 and 5 assessing by the AHRQ evaluation criteria for cross-sectional studies and NOS for cohort studies. The average total score was 6.20 (range from 4 to 9) for the cross-sectional studies and 7.29 (range from 7 to 8).

### Overall analyses

**Fig. 2:**
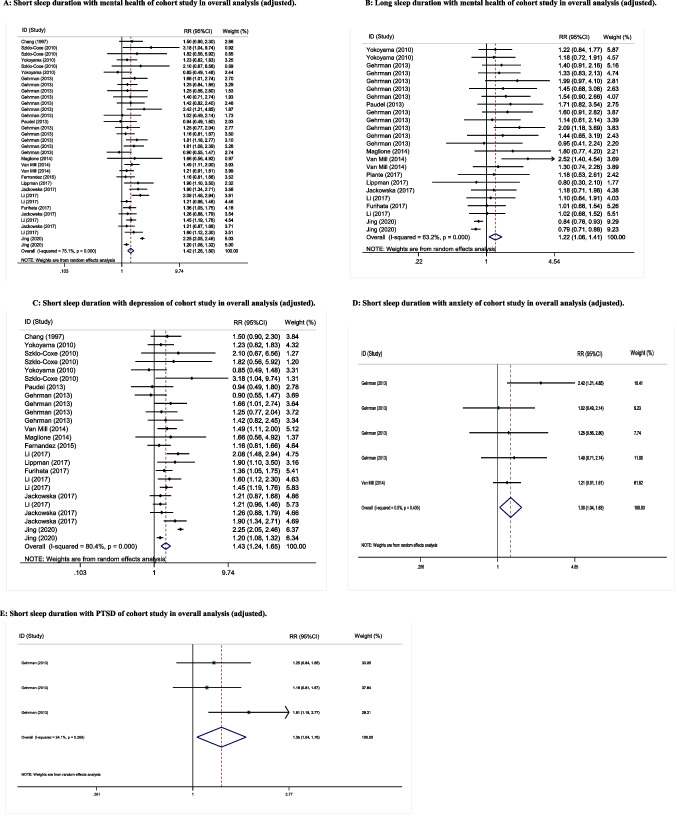
Overall analysis of sleep duration and mental disorders in cohort studies and cross-section studies with risk ratio (RR), odds ratio (OR), and 95% confidence interval (CI)

After compiling the findings from all qualified cohort and cross-sectional studies, both short and long sleep duration were statistically associated with the risk of mental disorders. According to the findings of the cohort studies (adjusted RR = 1.42, 95% CI: 1.26–1.60, *P* < .001, *I*^2^ = 37.6%, Tau^2^ = 0.014) and cross-sectional research, SSD negatively affected the risk of mental disorders (adjusted OR = 1.67, 95% CI: 1.57–1.77, *P* < .001, *I*^2^ = 79.7%, Tau^2^ = 0.060) (Fig. 2).

The overall analysis result also indicated that LSD had a negative effect on the likelihood of developing mental problems in the cohort (adjusted RR = 1.22, 95% CI: 1.06–1.41, *P* = .006, *I*^2^ = 63.2%, Tau^2^ = 0.055) and cross-sectional studies (adjusted OR = 1.20, 95% CI: 1.12–1.29, *P* < .001, *I*^2^ = 62.1%, Tau^2^ = 0.040).

### Cumulative and sensitivity analyses

The results of the combined analysis of the included researches were remarkably similar, and the tendency tended to hold in both cohort and cross-sectional investigations. Sensitivity analyses revealed no significant effect on any single study on overall effect-size estimates in the cohort cross-sectional studies.

### Publication bias

**Fig. 3:**
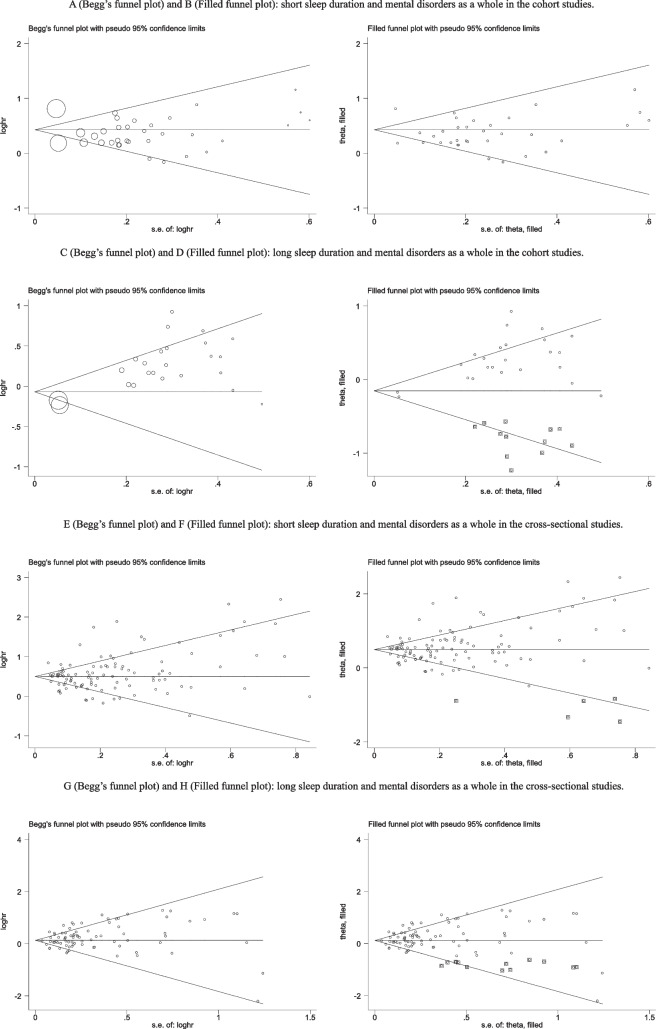
Begg’s and filled funnel plots for sleep duration and mental disorders

For the relationship between sleep duration and mental disorders, see Fig. 3 for Begg’s funnel plot of publication bias. In the cohort studies, no publication bias was found using Egger’s test for SSD (Coef. = −0.77, 95% CI: −1.90 to 0.36, *P* = .176), yet strong evidence of publication bias for LSD (Coef. = 2.00, 95% CI: 1.44 to 2.57, *P* = .000). Additional filled funnel plots revealed that 12 studies may have been omitted to make the LSD plot symmetrical because of publication bias. Effect size estimates for the relationship between LSD and mental disorders remained statistically significant after controlling for this potentially absent research.

In the cross-sectional studies, Egger’s test found that there was no evidence of publication bias for SSD with mental health (Coef. = 0.26, 95% CI: −0.47 to 0.99, *P* = .485). However, strong evidence of publication bias for LSD with mental disorders (Coef. = 0.64, 95% CI: 0.088 to 1.193, *P* = .024). And additional filled funnel plots revealed that there were 12 potentially missing studies to make the LSD plot more symmetrical.

### Subgroup analyses

To further analyze the heterogeneity between the included studies, a series of subgroup analyses were performed depending on the baseline data. Notably, the damaging effect of SSD on mental illness was consistent across subgroup analyses in both cohort and cross-sectional studies (Tables 2 and 3). However, significant heterogeneity was found in the results of LSD in both cohort and case-control studies, including different kinds of mental disorders, gender, age, ascertainment of sleep duration, career, and follow-up intervals.

**Table 2:** Overall and subgroup analyses of short and long sleep duration with mental disorder of adults in the cohort studies

Group	Number of qualified observations	Short sleep duration			Long sleep duration		
		RR (95% CI); *P*	*I*^2^	Tau^2^	RR (95% CI); *P*	*I*^2^	Tau^2^
Overall analyses							
Mental disorder (unadjusted)	10/7	1.44 (1.27–1.63); < .001	37.6%	0.014	1.30 (1.10–1.54); .002	0.0%	0.000
Mental disorder (adjusted)	36/24	1.42 (1.26–1.60); < .001	75.1%	0.071	1.22 (1.06–1.41); .006	63.2%	0.055
Subgroup analyses based on adjusted mental disorder							
By mental health							
Depression	25/17	1.43 (1.24–1.65); < .001	80.4%	0.082	1.15 (0.98–1.34); .088	63.4%	0.045
Anxiety	5/3	1.30 (1.04–1.63); .002	0.0%	0.000	1.37(0.93–2.03); .114	0.0%	0.000
PTSD	3/4	1.35 (1.04–1.76); .022	24.1%	0.013	1.44 (1.12–1.86); .005	0.0%	0.000
By gender							
Male	2/1	1.26 (0.81–1.96); .314	23.3%	0.026	1.71 (0.82–3.55); .150	*	0.000
Female	2/2	1.37 (1.07–1.76); < .001	0.0%	0.000	1.19 (0.71–1.99); .499	29.9%	0.050
Both genders	32/21	1.45 (1.28–1.64); .012	70.6%	0.076	1.21 (1.04–1.41); .012	65.1%	0.055
By age							
46–59	2/1	1.33 (1.11–1.59); .002	34.6%	0.006	1.02 (0.68–1.53); .923	*	0.000
> 60	14/11	1.46 (1.19–1.80); < .001	87.5%	0.112	0.96 (0.84–1.10); .574	42.4%	0.014
By country							
Developed	30/20	1.37 (1.26–1.49); < .001	0.1%	0.000	1.37 (1.21–1.56); < .001	0.0%	0.000
Developing	6/4	1.44 (1.28–1.61); .002	94.7%	0.120	0.83 (0.77–0.89); < .001	0.0%	0.000
By career							
General population	23/14	1.46 (1.26–1.70); < .001	82.0%	0.084	1.08 (0.92–1.26); .353	60.0%	0.033
Military personnel	12/10	1.37 (1.19–1.58); < .001	0.0%	0.000	1.47 (1.22–1.78); < .001	0.0%	0.000
By ascertainment of sleep duration							
Subjective method	30/19	1.44 (1.27–1.63); < .001	77.5%	0.073	1.20 (1.04–1.39); .015	65.0%	0.053
Objective method	6/2	1.29 (0.98–1.70); .070	4.2%	0.000	1.54 (0.98–2.42); .064	0.0%	0.000
By follow-up (years)							
<5	14/8	1.42 (1.24–1.63); < .001	81.0%	0.094	1.28 (1.06–1.54); .011	5.7%	0.004
≥5	22/16	1.43 (1.22–1.68); < .001	36.3%	0.020	1.18 (0.99–1.39); .059	64.5%	0.048
Sleep duration analysis							
≤ 5 h	4	1.64 (1.06–2.56); .027	37.2%	0.076	─	─	─
≤ 6 h	26	1.46 (1.27–1.69); < .001	69.7%	0.074	─	─	─
≤ 7 h	33	1.42 (1.26–1.60); < .001	75.8%	0.071	─	─	─
≥ 8 h	24	─	─	─	1.22 (1.06–1.41); .006	63.2%	0.055
≥ 9 h	8	─	─	─	1.20 (0.98–1.47); .080	13.9%	0.012
≥ 10 h	3	─	─	─	1.54 (0.98–2.44); .062	51.1%	0.083

*RR* risk ratio, *95% CI* 95% confidence interval, *PTSD* post-traumatic stress disorder

*Data are not available

**Table 3:** Overall and subgroup analyses of short and long sleep duration with mental disorder of adults in the cross-sectional studies

Group	Number of qualified observations	Short sleep duration			Long sleep duration		
		OR (95% CI); *P*	*I*^2^	Tau^2^	OR (95% CI); *P*	*I*^2^	Tau^2^
Overall analyses							
Mental disorder (unadjusted)	50/39	1.81 (1.67–1.95); < .001	83.9%	0.052	1.39 (1.25–1.56); < .001	86.3%	0.089
Mental disorder (adjusted)	107/81	1.67 (1.57–1.77); < .001	79.7%	0.060	1.20 (1.12–1.29); < .001	62.1%	0.040
Subgroup analyses based on adjusted mental disorder							
By mental health							
Depression	63/50	1.66 (1.55–1.77); < .001	76.0%	0.042	1.24 (1.15–1.35); < .001	66.9%	0.041
Anxiety	11/4	1.51 (1.21–1.89); < .001	84.1%	0.089	0.80 (0.58–1.09); .150	0.0%	0.000
BD	3/3	1.59 (0.84–3.02); .154	0.0%	0.000	0.60 (0.06–5.79); .658	73.2%	2.914
Phobia	4/4	1.89 (1.16–3.07); .010	55.6%	0.118	1.22 (0.79–1.88); .367	34.9%	0.064
PTSD	6/4	1.92 (1.21–3.03); .005	69.7%	0.214	1.70 (0.99–2.92); .054	65.4%	0.193
OCD	3/3	2.13 (1.24–3.66); .006	36.3%	0.086	0.89 (0.43–1.84); .756	0.0%	0.000
SA	3/*	6.14 (4.63–8.13); < .001	0.0%	0.000	*	*	*
SI	7/7	1.32 (1.14–1.53); < .001	28.5%	0.010	1.10 (0.86–1.40); .461	30.6%	0.028
PD	2/1	1.65 (0.72–3.80); .240	0.0%	0.000	1.04 (0.11–9.99); .973	*	0.000
By gender							
Male	13/9	1.62 (1.37–1.91); < .001	79.1%	0.066	1.23 (1.08–1.40); .002	0.0%	0.000
Female	15/11	1.63(1.45–1.85); < .001	77.0%	0.034	1.19 (1.04–1.37); .013	58.6%	0.028
Both genders	79/61	1.69 (1.56–1.82); < .001	80.5%	0.072	1.20 (1.10–1.31); < .001	66.6%	0.050
By age							
46–59	3/3	2.03 (1.19–3.47); .010	93.3%	0.207	1.42 (0.89–2.26); .138	74.3%	0.124
> 60	11/11	1.43 (1.19–1.71); < .001	74.7%	0.059	1.41 (1.23–1.61); < .001	29.5%	0.014
By country							
Developed	72/56	1.69 (1.53–1.85); < .001	75.1%	0.097	1.18 (1.08–1.29); < .001	54.1%	0.041
Developing	35/25	1.67 (1.54–1.81); < .001	84.9%	0.042	1.23 (1.11–1.37); < .001	73.6%	0.047
By career							
General population	82/69	1.64 (1.53–1.75); < .001	82.8%	0.060	1.23 (1.14–1.32); < .001	61.2%	0.040
Health care worker	12/2	1.76 (1.45–2.12); < .001	49.9%	0.052	1.26 (0.85–1.88); .253	0.0%	0.000
Military personnel	11/4	2.05 (1.43–2.95); < .001	59.3%	0.199	2.37 (1.33–4.23); .003	0.0%	0.000
By AHRQ							
< 5	6/4	3.01 (1.51–6.05); .002	75.9%	0.511	1.54 (0.95–2.51); .083	0.0%	0.000
≥ 5	101/77	1.65 (1.55–1.76); < .001	80.1%	0.059	1.20 (1.12–1.28); < .001	63.5%	0.041
Sleep duration analysis							
≤ 5 h	29	2.21 (1.84–2.66); < .001	78.4%	0.167	─	─	─
≤ 6 h	82	1.75 (1.62–1.90); < .001	80.7%	0.072	─	─	─
≤ 7 h	101	1.68 (1.57–1.79); < .001	80.4%	0.062	─	─	─
≥ 8 h	76	─	─	─	1.21 (1.13–1.30); < .001	61.4%	0.042
≥ 9 h	53	─	─	─	1.29 (1.19–1.39); < .001	56.8%	0.033
≥ 10 h	4	─	─	─	1.63 (1.27–2.08); < .001	0.0%	0.000

*RR* risk ratio, *95% CI* 95% confidence interval, *BD* bipolar disorder, *OCD* obsessive-compulsive disorder, *SA* suicide attempt, *SI* suicidal ideation, *PD* panic disorder, *PTSD* post-traumatic stress disorder

*Data are not available

SSD was statistically associated with depression risk (adjusted RR = 1.43, 95% CI: 1.24–1.65, *P* < .001, *I*^2^ = 37.6%, Tau^2^ = 0.014), anxiety risk (adjusted RR = 1.30, 95% CI: 1.04–1.63, *P* = .002, *I*^2^ = 0.0%, Tau^2^ = 0.000), and PTSD risk (adjusted RR = 1.35, 95% CI: 1.04–1.76, *P* = .022, *I*^2^ = 24.1%, Tau^2^ = 0.013) in the cohort studies (two-sample *Z*-test *P* = .241 for depression vs. anxiety, *P* = .353 for depression vs. PTSD, and *P* = .415 for anxiety vs. PTSD). LSD has not been proved to be a risk factor for depression and anxiety, although statistical results show that it was a deleterious factor for PTSD.

In the included cohort studies, there was a statistically significant difference between SSD and mental health in females (adjusted RR = 1.37, 95% CI: 1.07–1.76, *P* < .001, *I*^2^ = 0.0%, Tau^2^ = 0.000). No such association is found for males (adjusted RR = 1.26, 95% CI: 0.81–1.96, *P* = .314, *I*^2^ = 23.3%, Tau^2^ = 0.026) (two-sample *Z*-test *P* = .373). We found no evidence that long sleep duration is a risk factor for mental health.

The included cohort studies were divided into developing and developed countries. Subgroup analysis demonstrated statistical significance of SSD for mental disorders both in developing (adjusted RR = 1.44, 95% CI: 1.28–1.61, *P* = .002, *I*^2^ = 94.7%, Tau^2^ = 0.120) and developed countries (adjusted RR = 1.37, 95% CI: 1.26–1.49, *P* < .001, *I*^2^ = 0.1%, Tau^2^ = 0.000) (two-sample *Z*-test *P* = .246). Similarly, this relationship also held true for the LSD group.

Based on available age data, the population was divided into middle-aged (46–59 years) and elderly (≥ 60) groups. There was a statistically significant difference between SSD and mental disorders, both in middle-aged (adjusted RR = 1.33, 95% CI: 1.11–1.59, *P* = .002, *I*^2^ = 34.6%, Tau^2^ = 0.006) and elderly populations (adjusted RR = 1.46, 95% CI: 1.18–1.80, *P* < .001, *I*^2^ = 87.5%, Tau^2^ = 0.012) (two-sample *Z*-test *P* = .255) in the cohort studies. However, this statistical difference did not hold true in the LSD group.

Prominent differences were found both in general population (adjusted RR = 1.46, 95% CI: 1.26–1.70, *P* < .001, *I*^2^ = 82.0%, Tau^2^ = 0.084) and military personnel (adjusted RR = 1.37, 95% CI: 1.19–1.58, *P* < .001, *I*^2^ = 0.0%, Tau^2^ = 0.000) in cohort studies. There was a significant difference between LSD and mental disorders in military personnel (adjusted RR = 1.47, 95% CI: 1.22–1.78, *P* < .001, *I*^2^ = 0.0%, Tau^2^ = 0.000), but this difference was not significant in the general population.

Based on the ascertainment of sleep duration, we found a significant difference between the SSD and mental disorders in subjective method (adjusted RR = 1.44, 95% CI: 1.27–1.63, *P* < .001, *I*^2^ = 77.5%, Tau^2^ = 0.073). However, this relationship was not observed when objective methods (adjusted RR = 1.29, 95% CI: 0.98–1.70, *P* = .070, *I*^2^ = 4.2%, Tau^2^ = 0.000). Furthermore, LSD was identified as a risk factor for mental disorders when subjective methods were employed to measure sleep duration (adjusted RR = 1.20, 95% CI: 1.04–1.39, *P* = .015, *I*^2^ = 65.0%, Tau^2^ = 0.053), but not with objective methods (adjusted RR =1.54, 95% CI: 0.98–2.42, *P* = .064, *I*^2^ = 0.0%, Tau^2^ = 0.000).

The deleterious effects of SSD on mental disorders were consistent and significant in the cohort study, regardless of the length of follow-up (< 5 years: adjusted RR = 1.42, 95% CI: 1.24–1.63, *P* < .001, *I*^2^ = 81.0%, Tau^2^ = 0.094; ≥ 5 years: adjusted RR = 1.43, 95% CI: 1.22–1.68, *P* < .001, *I*^2^ = 36.3%, Tau^2^ = 0.020). When follow-up was < 5 years (adjusted RR = 1.28, 95% CI: 1.06–1.54, *P* = .011, *I*^2^ = 5.7%, Tau^2^ = 0.004), there was a statistically significant difference between LSD and mental disorders, yet this statistical difference could not be established at follow-up ≥ 5 years (adjusted RR = 1.18, 95% CI: 0.99–1.39, *P* = .059, *I*^2^ = 64.5%, Tau^2^ = 0.048).

We performed a more specific subgroup analysis of sleep duration, and the results were consistent with results of the overall analysis, which SSD remaining an independent risk factor for psychological disturbances, whether ≤ 5 h (adjusted RR = 1.64, 95% CI: 1.06–2.56, *P* = .027, *I*^2^ = 37.2%, Tau^2^ = 0.076), ≤ 6 h (adjusted RR = 1.46, 95% CI: 1.27–1.69, *P* < .001, *I*^2^ = 69.7%, Tau^2^ = 0.074), or ≤ 7 h (adjusted RR = 1.42, 95% CI: 1.26–1.60, *P* < .001, *I*^2^ = 75.8%, Tau^2^ = 0.071) (two-sample *Z*-test *P* = .311 for ≤ 5 h vs. ≤ 6 h and *P* = .385 for ≤ 6 h vs. ≤ 7 h). LSD as an independent risk factor for psychological disorders is not stable, and statistical results ≥ 9 h (adjusted RR = 1.20, 95% CI: 1.06–1.41, *P* = .006, *I*^2^ = 13.9%, Tau^2^ = 0.012) and ≥ 10 h (adjusted RR = 1.54, 95% CI: 0.98–2.44, *P* = .062, *I*^2^ = 51.1%, Tau^2^ = 0.083) (two-sample *Z*-test *P* = .448 for ≥ 8 h vs. ≥ 9 h and *P* = .044 for ≥ 9 h vs. ≥ 10 h) do not support the theory of overall analysis.

The overall and subgroup analysis of the cohort studies suggests that SSD is an independent risk factor for mental disorders. However, the results of subgroup analysis do not support that LSD is also a risk factor for psychological disorders.

Given the high heterogeneity of the results presented in the overall analysis of the relationship between sleep duration and mental disorders in cross-sectional studies, we correspondingly conducted a series of subgroup analyses to explore the heterogeneity. The results indicated that SSD remains an independent risk factor for psychological disturbances, both in the overall and subgroup analysis.

## Discussion

This is the comprehensive meta-analysis to date that explores the relationship between sleep duration and psychological disorders in adults. The findings show that SSD among women increases the risk of developing psychological disorders. However, the association between LSD and mental disorders requires further validation. In addition, different types of psychological disorders, gender, methods of measuring sleep duration, baseline age, and follow-up intervals are the possible causes of heterogeneity among studies. Our findings further strengthen the evidence for an association between short sleep duration and mental health. A meta-analysis of seven cohort studies by Zhai and colleagues ^74^ found that long and short sleep durations increase the risk of depression in adults. This meta-analysis examined the relationship between sleep duration and psychological disorders by analyzing 52 research articles, including 14 cohort studies and 38 cross-sectional studies. These studies covered various types of psychological disorders such as depression, anxiety, PTSD, phobia, and suicidal attempts. The analysis combined effect size estimates from these publications, which involved a total of 1,406,197 adults, to determine the association between sleep duration and mental health. Despite consistently marginal significance in overall and subgroup analyses, the findings extended those of Zhai et al. revealing a negative association between short sleep duration (SSD) and mental health [73]. Evidence based on overall and subgroup analyses does not adequately demonstrate LSD as a risk factor for the development of psychological disorders, which contradicts the findings of Zhai and colleagues [73].

The inconsistencies in the above results could derive from several factors. First, the number of included articles. We included twice as many cohort studies as Zhai and his colleagues [73] and also different types of mental disorders. LSD was found to be a risk factor for psychological disorders development for most articles included in this meta-analysis.

The second factor was the different types of study designs of the included studies. Cross-sectional studies show the correlation between variables but do not show whether one variable precedes another in the causal chain [74]. Although informative, it is not possible to infer causality from these studies. Longitudinal designs provide stronger evidence. SSD was a constant independent predictor of psychological morbidity in both cross-sectional and cohort studies. Although there is a strong relationship between LSD and psychological disorders in cross-sectional studies, LSD should be included in cohort studies.

The third factor may be significant heterogeneity across studies. Subgroup analyses and meta-regression analyses identified different psychiatric disorders, gender, level of economic development, method of sleep monitoring, baseline age, and follow-up interval as potential sources of heterogeneity among studies. This study recommends future large-scale, well-designed cohort studies to give reliable estimates. We found high heterogeneity between LSD and the development of psychological disorders in adults regardless of study type.

In contrast, for SSD, heterogeneity was low in both cross-sectional and cohort studies. Accordingly, this meta-analysis suggests that in addition to methodological heterogeneity (e.g., study design), clinical heterogeneity such as different baseline characteristics (e.g., age, sex ratio, and type of psychological disorders) of the study population may be the source of this difference. Notably, residual confounders were potentially inadequately corrected for incompletely measured or unmeasured clinical covariates. Consequently, translating LSD as a predictor of mental disorders into clinical practice should be done with caution.

Sleep is crucial for the health and well-being of a person’s life. Adequate sleep is necessary for physiological recovery. However, lack of sleep is increasingly a public health problem. The relationship between the sleep state and the development of mental disorders remains to be elucidated. Nevertheless, several theories have been proposed to explain this phenomenon.

First, inflammation is one of the dominant factors that causes depression [75]. Studies suggest that elevated inflammatory cytokines such as CRP and IL6 are strongly associated with lack of sleep and poor sleep quality [76–78]. Persistent short sleep duration leads to elevated levels of IL-1-like and IL-2-like activity, and this increase is independent of the circadian rhythm of cortisol [79]. At the same time, as the “dose'” of short sleep duration progressively increases over 4 nights, there is evidence of cumulative increase of CRP [80].

Another factor that can cause depression is SSD which activates the hypothalamic-pituitary-adrenal axis. Research evidence suggests that over-activation of the hypothalamic-pituitary-adrenal axis causes depression [81, 82]. Third, physical and psychological fatigue during the day resulting from poor sleep at night potentially disrupts circadian rhythms and causes hormonal changes, causing depression [83–85]. Melatonin is a pleiotropic molecule that can alleviate depression. A good night’s sleep, including the appropriate sleep duration, increases melatonin levels in the body [86, 87].

Fourth, perceived stress has been reported as a risk factor for depression. Individuals with short sleep duration may be less rested and have higher stress severity [88]. Perceived stress has been reported to be a risk factor for depressive symptoms [89]. Poor sleep quality caused by persistent short sleep duration can lead to diminished cognition, mood, and physical activity, which can exacerbate depressive symptoms [17, 48, 86].

Although the literature we have included has limited coverage of gender differences, our preliminary findings suggested that depressive symptoms are more prevalent in females with SSD compared to males, although this association was not statistically significant in males. Reasons for females to be more prone to depression include the direct effect of follicular hormones [90, 91]. The hypothalamic-pituitary-adrenal (HPA) axis, which regulates stress, tends to be more dysfunctional in women [92] affecting the interaction between follicular hormones and HPA regulation [93].

It has been suggested that dysregulation of the 31-hydroxytryptaminergic system may be a potential mechanism underlying the observed sex-specific relationship between sleep symptoms and depression [94]. Furthermore, most women experience premenstrual symptoms throughout their lives and about one in five report severe symptoms including depression [95]. Females also respond and adapt differently to stress. dolescent girls tend to be more concerned with stressful emotions and mental distress [96].

It is therefore important to include sleep duration when opting for appropriate interventions and monitoring treatments for psychological disorders. Both good sleep and positive mental health indicate a healthy lifestyle [48]. However, further research is necessary to clarify the effect of sleep duration on mental well-being to determine if there is a cause-and-effect relationship between sleep duration and mental health. There were several limitations in this study. First, in most studies, sleep duration was evaluated using subjective questionnaires. Therefore, future studies should objectively measure sleep duration. Second, our analyses did not find sufficient evidence to support LSD as an independent predictor of mental disorders due to the limited available data. To gain a better understanding of whether or not LSD is indeed an independent risk factor for mental disorders, more high-quality studies are required. Only six articles explicitly considered obstructive sleep apnea (OSA) as an adjustment factor. Future research should focus on exploring the effects of the interaction between sleep disorders, including OSA, and sleep duration on mental health. Several subgroup analyses were conducted to examine the heterogeneity among studies in the overall analysis. However, significant heterogeneity was observed within various subgroups, which make it challenging to interpret the combined effect size estimates accurately.

## Conclusion

Our findings suggest that SSD is an independent predictor of developing mental disorders, particularly anxiety and depression. Despite our results, tThe effect of LSD on psychological disorders requires further validation. 

### Supplementary information

ESM 1 (DOCX 61 kb)

## Data Availability

My manuscript has no associated data.
