# Supplementary material for: Association of sleep duration and risk of mental disorder: a systematic review and meta-analysis
Source: Sleep Breath. 2023 Aug 29;28(1):261–80. doi: 10.1007/s11325-023-02905-1 (PMC10954977; doi:10.1007/s11325-023-02905-1)
Supplement: Supplementary file 1 — (DOCX 61 kb) [file 11325_2023_2905_MOESM1_ESM.docx]

**Supplementary Online Content**

eTable 1 Reporting checklist for systematic review (with a meta-analysis).

eTable 2. MOOSE Checklist for Meta-analyses of Observational Studies

eTable 3. Search Terms and Number of Records

eTable 4. AHRQ for assessing the quality of all cross-sectional studies involved in this meta-analysis.

etable 5. The Newcastle-Ottawa Scale (NOS) for assessing the quality of all cohort studies involved in this meta-analysis.

# **eTable 1. Reporting checklist for systematic review (with or without a meta-analysis).**

Based on the PRISMA guidelines.

## Instructions to authors

Complete this checklist by entering the page numbers from your manuscript where readers will find each of the items listed below.

Your article may not currently address all the items on the checklist. Please modify your text to include the missing information. If you are certain that an item does not apply, please write "n/a" and provide a short explanation.

Upload your completed checklist as an extra file when you submit to a journal.

In your methods section, say that you used the PRISMAreporting guidelines, and cite them as:

Page MJ, McKenzie JE, Bossuyt PM, Boutron I, Hoffmann TC, Mulrow CD, Shamseer L, Tetzlaff JM, Akl EA, Brennan SE, Chou R, Glanville J, Grimshaw JM, Hróbjartsson A, Lalu MM, Li T, Loder EW, Mayo-Wilson E, McDonald S, McGuinness LA, Stewart LA, Thomas J, Tricco AC, Welch VA, Whiting P, Moher D. The PRISMA 2020 statement: An updated guideline for reporting systematic reviews

|  |  | Reporting Item | Page Number |
| --- | --- | --- | --- |
| **Title** |  |  |  |
| Title | [#1](https://www.goodreports.org/reporting-checklists/prisma/info/#1) | Identify the report as a systematic review | 1 |
| **Abstract** |  |  |  |
| Abstract | [#2](https://www.goodreports.org/reporting-checklists/prisma/info/#2) | Report an abstract addressing each item in the PRISMA 2020 for Abstracts checklist | 2-3 |
| **Introduction** |  |  |  |
| Background/rationale | [#3](https://www.goodreports.org/reporting-checklists/prisma/info/#3) | Describe the rationale for the review in the context of existing knowledge | 4-6 |
| Objectives | [#4](https://www.goodreports.org/reporting-checklists/prisma/info/#4) | Provide an explicit statement of the objective(s) or question(s) the review addresses | 5-6 |
| **Methods** |  |  |  |
| Eligibility criteria | [#5](https://www.goodreports.org/reporting-checklists/prisma/info/#5) | Specify the inclusion and exclusion criteria for the review and how studies were grouped for the syntheses | 6-7 |
| Information sources | [#6](https://www.goodreports.org/reporting-checklists/prisma/info/#6) | Specify all databases, registers, websites, organisations, reference lists, and other sources searched or consulted to identify studies. Specify the date when each source was last searched or consulted | 5 |
| Search strategy | [#7](https://www.goodreports.org/reporting-checklists/prisma/info/#7) | Present the full search strategies for all databases, registers, and websites, including any filters and limits used | 5-6, eTable3 |
| Selection process | [#8](https://www.goodreports.org/reporting-checklists/prisma/info/#8) | Specify the methods used to decide whether a study met the inclusion criteria of the review, including how many reviewers screened each record and each report retrieved, whether they worked independently, and, if applicable, details of automation tools used in the process | 6-7  Figure 1 |
| Data collection process | [#9](https://www.goodreports.org/reporting-checklists/prisma/info/#9) | Specify the methods used to collect data from reports, including how many reviewers collected data from each report, whether they worked independently, any processes for obtaining or confirming data from study investigators, and, if applicable, details of automation tools used in the process | 7 |
| Data items | [#10a](https://www.goodreports.org/reporting-checklists/prisma/info/#10a) | List and define all outcomes for which data were sought. Specify whether all results that were compatible with each outcome domain in each study were sought (for example, for all measures, time points, analyses), and, if not, the methods used to decide which results to collect | 7  Table 1 |
| Data items | [#10b](https://www.goodreports.org/reporting-checklists/prisma/info/#10b) | List and define all other variables for which data were sought (such as participant and intervention characteristics, funding sources). Describe any assumptions made about any missing or unclear information | Table 1 |
| Study risk of bias assessment | [#11](https://www.goodreports.org/reporting-checklists/prisma/info/#11) | Specify the methods used to assess risk of bias in the included studies, including details of the tool(s) used, how many reviewers assessed each study and whether they worked independently, and, if applicable, details of automation tools used in the process | 7-8 |
| Effect measures | [#12](https://www.goodreports.org/reporting-checklists/prisma/info/#12) | Specify for each outcome the effect measure(s) (such as risk ratio, mean difference) used in the synthesis or presentation of results | 8 |
| Synthesis methods | [#13a](https://www.goodreports.org/reporting-checklists/prisma/info/#13a) | Describe the processes used to decide which studies were eligible for each synthesis (such as tabulating the study intervention characteristics and comparing against the planned groups for each synthesis (item #5)) | 7-8  Table 1 |
| Synthesis methods | [#13b](https://www.goodreports.org/reporting-checklists/prisma/info/#13b) | Describe any methods required to prepare the data for presentation or synthesis, such as handling of missing summary statistics or data conversions | 7-8 |
| Synthesis methods | [#13c](https://www.goodreports.org/reporting-checklists/prisma/info/#13c) | Describe any methods used to tabulate or visually display results of individual studies and syntheses | 7-8 |
| Synthesis methods | [#13d](https://www.goodreports.org/reporting-checklists/prisma/info/#13d) | Describe any methods used to synthesise results and provide a rationale for the choice(s). If meta-analysis was performed, describe the model(s), method(s) to identify the presence and extent of statistical heterogeneity, and software package(s) used | 8 |
| Synthesis methods | [#13e](https://www.goodreports.org/reporting-checklists/prisma/info/#13e) | Describe any methods used to explore possible causes of heterogeneity among study results (such as subgroup analysis, meta-regression) | 8 |
| Synthesis methods | [#13f](https://www.goodreports.org/reporting-checklists/prisma/info/#13f) | Describe any sensitivity analyses conducted to assess robustness of the synthesised results | 9 |
| Reporting bias assessment | [#14](https://www.goodreports.org/reporting-checklists/prisma/info/#14) | Describe any methods used to assess risk of bias due to missing results in a synthesis (arising from reporting biases) | 9 |
| Certainty assessment | [#15](https://www.goodreports.org/reporting-checklists/prisma/info/#15) | Describe any methods used to assess certainty (or confidence) in the body of evidence for an outcome | 9 |
| **Results** |  |  |  |
| Study selection | [#16a](https://www.goodreports.org/reporting-checklists/prisma/info/#16a) | Describe the results of the search and selection process, from the number of records identified in the search to the number of studies included in the review, ideally using a flow diagram (http://www.prisma-statement.org/PRISMAStatement/FlowDiagram) | Figure1 |
| Study selection | [#16b](https://www.goodreports.org/reporting-checklists/prisma/info/#16b) | Cite studies that might appear to meet the inclusion criteria, but which were excluded, and explain why they were excluded | Figure1 |
| Study characteristics | [#17](https://www.goodreports.org/reporting-checklists/prisma/info/#17) | Cite each included study and present its characteristics | 9-10 |
| Risk of bias in studies | [#18](https://www.goodreports.org/reporting-checklists/prisma/info/#18) | Present assessments of risk of bias for each included study | eTable4&5 |
| Results of individual studies | [#19](https://www.goodreports.org/reporting-checklists/prisma/info/#19) | For all outcomes, present for each study (a) summary statistics for each group (where appropriate) and (b) an effect estimate and its precision (such as confidence/credible interval), ideally using structured tables or plots | Table 2 |
| Results of syntheses | [#20a](https://www.goodreports.org/reporting-checklists/prisma/info/#20a) | For each synthesis, briefly summarise the characteristics and risk of bias among contributing studies | 10-11, Table 1 |
| Results of syntheses | [#20b](https://www.goodreports.org/reporting-checklists/prisma/info/#20b) | Present results of all statistical syntheses conducted. If meta-analysis was done, present for each the summary estimate and its precision (such as confidence/credible interval) and measures of statistical heterogeneity. If comparing groups, describe the direction of the effect | 10-16, Figure 2 |
| Results of syntheses | [#20c](https://www.goodreports.org/reporting-checklists/prisma/info/#20c) | Present results of all investigations of possible causes of heterogeneity among study results | Table 2 |
| Results of syntheses | [#20d](https://www.goodreports.org/reporting-checklists/prisma/info/#20d) | Present results of all sensitivity analyses conducted to assess the robustness of the synthesised results | 10-16 |
| Risk of reporting biases in syntheses | [#21](https://www.goodreports.org/reporting-checklists/prisma/info/#21) | Present assessments of risk of bias due to missing results (arising from reporting biases) for each synthesis assessed | 11-12 |
| Certainty of evidence | [#22](https://www.goodreports.org/reporting-checklists/prisma/info/#22) | Present assessments of certainty (or confidence) in the body of evidence for each outcome assessed | 10-16 |
| **Discussion** |  |  |  |
| Results in context | [#23a](https://www.goodreports.org/reporting-checklists/prisma/info/#23a) | Provide a general interpretation of the results in the context of other evidence | 16-18 |
| Limitations of included studies | [#23b](https://www.goodreports.org/reporting-checklists/prisma/info/#23b) | Discuss any limitations of the evidence included in the review | 18 |
| Limitations of the review methods | [#23c](https://www.goodreports.org/reporting-checklists/prisma/info/#23c) | Discuss any limitations of the review processes used | 18-19 |
| Implications | [#23d](https://www.goodreports.org/reporting-checklists/prisma/info/#23d) | Discuss implications of the results for practice, policy, and future research | 19-20 |
| **Other information** |  |  |  |
| Registration and protocol | [#24a](https://www.goodreports.org/reporting-checklists/prisma/info/#24a) | Provide registration information for the review, including register name and registration number, or state that the review was not registered | 6 |
| Registration and protocol | [#24b](https://www.goodreports.org/reporting-checklists/prisma/info/#24b) | Indicate where the review protocol can be accessed, or state that a protocol was not prepared | 6 |
| Registration and protocol | [#24c](https://www.goodreports.org/reporting-checklists/prisma/info/#24c) | Describe and explain any amendments to information provided at registration or in the protocol | 6 |
| Support | [#25](https://www.goodreports.org/reporting-checklists/prisma/info/#25) | Describe sources of financial or non-financial support for the review, and the role of the funders or sponsors in the review | 22 |
| Competing interests | [#26](https://www.goodreports.org/reporting-checklists/prisma/info/#26) | Declare any competing interests of review authors | 22 |
| Availability of data, code, and other materials | [#27](https://www.goodreports.org/reporting-checklists/prisma/info/#27) | Report which of the following are publicly available and where they can be found: template data collection forms; data extracted from included studies; data used for all analyses; analytic code; any other materials used in the review | 22 |

None The PRISMA checklist is distributed under the terms of the Creative Commons Attribution License CC-BY. This checklist can be completed online using <https://www.goodreports.org/>, a tool made by the [EQUATOR Network](https://www.equator-network.org) in collaboration with Penelope

**eTable 2 MOOSE Checklist for Meta-analyses of Observational Studies**

| **Item No** | **Recommendation** | **Reported on Page No** |
| --- | --- | --- |
| **Reporting of background should include** | | |
| 1 | Problem definition | 2-3 |
| 2 | Hypothesis statement | 2-3 |
| 3 | Description of study outcome(s) | 2-3 |
| 4 | Type of exposure or intervention used | 2-3 |
| 5 | Type of study designs used | 2-3 |
| 6 | Study population | 2-3 |
| **Reporting of search strategy should include** | | |
| 7 | Qualifications of searchers (eg. librarians and investigators) | 5 |
| 8 | Search strategy, including time period included in the synthesis and key words | eTabe3 |
| 9 | Effort to include all available studies, including contact with authors | 5-6 |
| 10 | Databases and registries searched | 5 |
| 11 | Search software used, name and version, including special features used (eg. explosion) | 6 |
| 12 | Use of hand searching (eg. reference lists of obtained articles) | 5 |
| 13 | List of citations located and those excluded, including justification | 6 |
| 14 | Method of addressing articles published in languages other than English | 5-6 |
| 15 | Method of handling abstracts and unpublished studies | 5-6 |
| 16 | Description of any contact with authors | NA |
| **Reporting of methods should include** | | |
| 17 | Description of relevance or appropriateness of studies assembled for assessing the hypothesis to be tested | 6 |
| 18 | Rationale for the selection and coding of data (eg. sound clinical principles or convenience) | 6-7 |
| 19 | Documentation of how data were classified and coded (eg. multiple raters, blinding and interrater reliability) | 6-7 |
| 20 | Assessment of confounding (eg. comparability of cases and controls in studies where appropriate) | 6-7 |
| 21 | Assessment of study quality, including blinding of quality assessors, stratification or regression on possible predictors of study results | 7 |
| 22 | Assessment of heterogeneity | 7-8 |
| 23 | Description of statistical methods (eg. complete description of fixed or random effects models, justification of whether the chosen models account for predictors of study results, dose-response models, or cumulative meta-analysis) in sufficient detail to be replicated | 7-8 |
| 24 | Provision of appropriate tables and graphics | Supplementary tables  Figures |
| **Reporting of results should include** | | |
| 25 | Graphic summarizing individual study estimates and overall estimate | Table 2, 9-16 |
| 26 | Table giving descriptive information for each study included | Table 1, 9-11 |
| 27 | Results of sensitivity testing (eg. subgroup analysis) | Table 1 |
| 28 | Indication of statistical uncertainty of findings | 12-16 |
| **Reporting of discussion should include** | | |
| 29 | Quantitative assessment of bias (eg. publication bias) | eTable 4&5 |
| 30 | Justification for exclusion (eg. exclusion of non-English language citations) | Figure 1 |
| 31 | Assessment of quality of included studies | eTable 4&5 |
| **Reporting of conclusions should include** | | |
| 32 | Consideration of alternative explanations for observed results | 16-17 |
| 33 | Generalization of the conclusions (ie. appropriate for the data presented and within the domain of the literature review) | 17-19 |
| 34 | Guidelines for future research | 19-20 |
| 35 | Disclosure of funding source | 21 |

.

**eTable 3. Search Terms and Number of Records**

| **Search Terms and Number of Records** |
| --- |
| **PubMed (N= 3256)** |
| (Sleep) OR (sleep duration) OR (sleep disorders) OR (sleep disordered) OR (bed time) OR (insomnia) OR (actigraphy sleep) OR (self-reported sleep) OR (drowse) OR (napping) OR (naps) OR (nap) OR (Siesta) OR (drowsiness) [Title]AND (mental health) OR (mental hygiene) OR (mental disorder) OR (mental status) OR (mental deficiency) OR (psychiatric disease) OR (psychiatric illness) OR (psychiatric disorder) OR (psychological symptoms) OR (obsession-compulsion) OR (obsessive-compulsive disorder) OR (obsessive behavior) OR (compulsive personality disorder) OR (depression) OR (depressive symptom) OR (emotional depression) OR (depressive disorder) OR (depressive syndrome) OR (anxiety disorder) OR (anxiety neuroses) OR (neurotic anxiety) OR (hostility) OR (phobic disorder) OR (phobic neuroses) OR (phobic anxiety) OR (paranoid ideation) OR (paranoid disorder) OR (suicide) [Title] |
| **Web of Science (N= 4465)** |
| TI=(Sleep OR "sleep duration" OR "sleep disorders" OR ''sleep disordered'' OR "bed time" OR "insomnia" OR "actigraphy sleep" OR "self-reported sleep" OR drowsy OR napping OR naps OR Siesta OR drowsiness) AND TI=("mental health" OR "mental hygiene" OR "mental disorder" OR "mental status" OR "mental deficiency" OR "psychiatric disease" OR "self-reported sleep" OR "psychiatric illness“ OR "psychiatric disorder" OR "psychological symptoms" OR "obsession-compulsion" OR "obsessive-compulsive disorder" OR "obsessive behavior" OR "compulsive personality disorder" OR depression OR "depressive symptom" OR "emotional depression" OR "depressive disorder" OR "depressive syndrome" OR "anxiety disorder" OR "anxiety neuroses" OR "neurotic anxiety" OR hostility OR "phobic disorder" OR "phobic neuroses" OR "phobic anxiety" OR "paranoid ideation" OR "paranoid disorder" OR suicide) |
| **EMBASE (N=5118)** |
| sleep: ti OR 'sleep dura tion': ti OR 'sleep disorders': ti OR 'sleep disordered': ti OR 'bed time': ti OR 'insomnia': ti OR 'ac tigraphy sleep': ti OR 'self-reported sleep': ti OR drowsy: ti OR napping: ti OR naps: ti OR nap: ti OR siesta: ti OR drowsiness: ti) AND ( 'mental health': ti OR 'mental hygiene': ti OR 'mental status': ti OR 'mental disorder': ti OR 'mental deficiency': ti OR 'psychiatric disease': ti OR 'psychiatric illness': ti OR 'psychiatric disorder': ti OR 'psychological symptoms': ti OR 'obsession-compulsion': ti OR 'obsessive-compulsive disorder': ti OR 'obsessive behavior': ti OR 'compulsive personality disorder': ti OR depression: ti OR 'depressive symptom': ti OR 'emo tional depression': ti OR 'depressive disorder': ti OR 'depressive syndrome': ti OR 'anxiety disorder': ti OR 'anxiety neuroses': ti OR 'neuro tic anxiety': ti OR hos tility: ti OR 'phobic disorder': ti OR 'phobic neuroses': ti OR 'phobic anxiety': ti OR 'paranoid idea tion': ti OR 'paranoid disorder': ti OR suicide: ti) |
| **Scopus (N= 4909)** |
| ((TITLE (sleep) OR TITLE ("sleep duration") OR TITLE ("sleep disorders") OR TITLE ("sleep disordered") OR TITLE ("bed time") OR TITLE (insomnia) OR TITLE ("actigraphy sleep") OR TITLE ("self-reported sleep") OR TITLE (drowse) OR TITLE (napping) OR TITLE (naps) OR TITLE (nap) OR TITLE (siesta) OR TITLE (drowsiness))) AND ((TITLE ("mental health") OR TITLE ("mental hygiene") OR TITLE ("mental disorder ") OR TITLE ("mental status") OR TITLE ("mental deficiency") OR TITLE ("psychiatric disease") OR TITLE ("psychiatric illness") OR TITLE ("psychiatric disorder") OR TITLE ("psychological symptoms") OR TITLE ("obsession-compulsion") OR TITLE ("obsessive-compulsive disorder") OR TITLE ("obsessive behavior") OR TITLE ("compulsive personality disorder") OR TITLE (depression) OR TITLE ("depressive symptom") OR TITLE ("emotional depression") OR TITLE ("depressive disorder") OR TITLE ("depressive syndrome") OR TITLE ("anxiety disorder") OR TITLE ("anxiety neuroses") OR TITLE ("neurotic anxiety") OR TITLE (hostility) OR TITLE ("phobic disorder") OR TITLE ("phobic neuroses") OR TITLE ("phobic anxiety") OR TITLE ("paranoid ideation") OR TITLE ("paranoid disorder") OR TITLE (suicide))) |

**eTable 4. AHRQ for assessing the quality of all cross-sectional studies involved in this meta-analysis.**

| **Year** | **First author** | **(1)** | **(2)** | **(3)** | **(4)** | **(5)** | **(6)** | **(7)** | **(8)** | **(9)** | **(10)** | **(11)** | **Total** |
| --- | --- | --- | --- | --- | --- | --- | --- | --- | --- | --- | --- | --- | --- |
| 2002 | Hidalgo | 1 | 1 | 1 | 0 | 1 | 0 | 0 | 0 | 0 | 0 | 0 | 4 |
| 2005 | John | 1 | 1 | 0 | 0 | 1 | 0 | 1 | 1 | 0 | 1 | 0 | 6 |
| 2008 | Paudel | 1 | 1 | 1 | 0 | 1 | 0 | 1 | 1 | 0 | 1 | 0 | 7 |
| 2010 | Yokoyama | 1 | 1 | 1 | 1 | 1 | 0 | 1 | 1 | 0 | 1 | 1 | 9 |
| 2010 | Park | 1 | 1 | 1 | 1 | 1 | 0 | 1 | 1 | 0 | 1 | 0 | 8 |
| 2010 | Wada | 1 | 1 | 0 | 1 | 1 | 0 | 1 | 1 | 0 | 1 | 0 | 7 |
| 2011 | Blasco-Fontecilla | 1 | 1 | 1 | 0 | 1 | 0 | 1 | 1 | 0 | 1 | 0 | 7 |
| 2011 | Chang | 1 | 1 | 1 | 0 | 1 | 0 | 1 | 1 | 0 | 1 | 0 | 7 |
| 2013 | Sakamoto | 1 | 1 | 0 | 0 | 1 | 0 | 1 | 1 | 0 | 1 | 0 | 6 |
| 2013 | Swinkels | 1 | 1 | 0 | 0 | 1 | 0 | 0 | 1 | 0 | 1 | 0 | 5 |
| 2014 | Taylor | 1 | 1 | 0 | 0 | 1 | 0 | 0 | 1 | 0 | 0 | 0 | 4 |
| 2015 | Furihata | 1 | 1 | 1 | 0 | 1 | 0 | 1 | 1 | 1 | 1 | 0 | 8 |
| 2015 | Grossi | 1 | 1 | 1 | 0 | 1 | 0 | 1 | 1 | 0 | 1 | 0 | 7 |
| 2015 | Lee | 1 | 1 | 0 | 0 | 1 | 0 | 1 | 1 | 1 | 1 | 0 | 7 |
| 2016 | Plante | 1 | 1 | 1 | 0 | 1 | 0 | 0 | 1 | 0 | 0 | 1 | 6 |
| 2017 | Furihata | 1 | 1 | 1 | 0 | 1 | 0 | 1 | 1 | 0 | 1 | 1 | 8 |
| 2017 | Lippman | 1 | 1 | 1 | 0 | 1 | 0 | 0 | 1 | 0 | 0 | 1 | 6 |
| 2017 | Mohan | 1 | 1 | 1 | 0 | 1 | 0 | 1 | 1 | 0 | 1 | 0 | 7 |
| 2017 | Supartini | 1 | 1 | 1 | 0 | 1 | 0 | 0 | 1 | 0 | 0 | 0 | 5 |
| 2017 | Thomas | 1 | 1 | 1 | 0 | 1 | 0 | 0 | 1 | 0 | 0 | 0 | 5 |
| 2017 | Wang | 1 | 1 | 1 | 0 | 1 | 0 | 1 | 1 | 0 | 1 | 0 | 7 |
| 2018 | Liu | 1 | 1 | 1 | 0 | 1 | 0 | 0 | 1 | 0 | 0 | 0 | 5 |
| 2018 | Peltzer | 1 | 1 | 1 | 0 | 1 | 0 | 1 | 1 | 0 | 1 | 0 | 7 |
| 2018 | Sullivan | 1 | 1 | 1 | 0 | 1 | 0 | 0 | 1 | 0 | 0 | 0 | 5 |
| 2018 | Sun | 1 | 1 | 1 | 0 | 1 | 0 | 1 | 1 | 0 | 1 | 0 | 7 |
| 2019 | Ibrahim | 1 | 1 | 1 | 0 | 1 | 0 | 1 | 1 | 0 | 1 | 0 | 7 |
| 2019 | Ouyang | 1 | 1 | 1 | 0 | 1 | 0 | 1 | 1 | 0 | 1 | 0 | 7 |
| 2020 | AI-Ajlouni | 1 | 1 | 1 | 0 | 1 | 0 | 1 | 1 | 0 | 1 | 0 | 7 |
| 2020 | Chen | 1 | 1 | 1 | 0 | 1 | 0 | 1 | 1 | 0 | 1 | 0 | 7 |
| 2020 | Jiang | 1 | 1 | 1 | 0 | 1 | 0 | 1 | 1 | 0 | 0 | 0 | 6 |
| 2020 | Lai | 1 | 1 | 1 | 0 | 1 | 0 | 1 | 1 | 0 | 0 | 0 | 6 |
| 2020 | Li | 1 | 1 | 1 | 0 | 1 | 0 | 1 | 1 | 0 | 0 | 0 | 6 |
| 2020 | Matsui | 1 | 0 | 1 | 0 | 1 | 0 | 1 | 1 | 0 | 0 | 0 | 5 |
| 2020 | Seow | 1 | 1 | 1 | 0 | 1 | 0 | 1 | 1 | 0 | 0 | 0 | 5 |
| 2020 | Simmons | 1 | 0 | 1 | 0 | 1 | 0 | 0 | 1 | 0 | 0 | 0 | 4 |
| 2020 | Tonon | 1 | 1 | 1 | 0 | 1 | 0 | 0 | 1 | 0 | 0 | 0 | 5 |
| 2020 | Tubbs | 1 | 0 | 1 | 0 | 1 | 0 | 0 | 1 | 0 | 0 | 0 | 4 |
| 2021 | Ko | 1 | 1 | 1 | 0 | 1 | 0 | 1 | 1 | 0 | 0 | 0 | 6 |
| 2022 | Ding | 1 | 1 | 1 | 0 | 1 | 0 | 1 | 1 | 0 | 1 | 0 | 7 |
| 2022 | Dong | 1 | 1 | 1 | 0 | 1 | 0 | 1 | 1 | 0 | 1 | 0 | 7 |
| 2022 | Luo | 1 | 1 | 1 | 0 | 1 | 0 | 1 | 0 | 0 | 0 | 0 | 5 |

Notes: (1) Define the source of information (survey, record review); (2) List inclusion and exclusion criteria for exposed and unexposed subjects (cases and controls) or refer to previous publications; (3) Indicate time period used for identifying patients; (4) Indicate whether or not subjects were consecutive if not population-based; (5) Indicate if evaluators of subjective components of study were masked to other aspects of the status of the participants; (6) Describe any assessments undertaken for quality assurance purposes (e.g. test/retest of primary outcome measurements); (7) Explain any patient exclusions from analysis; (8) Describe how confounding was assessed and/or controlled; (9) If applicable, explain how missing data were handled in the analysis; (10) Summarize patient response rates and completeness of data collection; (11) Clarify what follow-up, if any, was expected and the percentage of patients for which incomplete data or follow-up was obtained.

**etable 5.** **The Newcastle-Ottawa Scale (NOS) for assessing the quality of all cohort studies involved in this meta-analysis.**

| **First author** | **Published year** | **Representative of the exposed cohort** | **Selection of the non-exposed cohort** | **Ascertainment of exposed** | **Demonstration that outcome of interest was no present at start of study** | **Control for important cohort** | **Additional factors** | **Assessment of outcome** | **Follow up** | **Adequacy of follow up** | **Score** |
| --- | --- | --- | --- | --- | --- | --- | --- | --- | --- | --- | --- |
| Chang | 1997 | 1 | 1 | 1 | 1 | 1 | 0 | 1 | 1 | 0 | 7 |
| Szklo-Coxe | 2010 | 1 | 1 | 1 | 1 | 1 | 0 | 1 | 1 | 1 | 8 |
| Yokoyama | 2010 | 1 | 1 | 1 | 1 | 1 | 0 | 1 | 1 | 0 | 7 |
| Paudel | 2013 | 1 | 1 | 1 | 1 | 1 | 0 | 1 | 1 | 0 | 7 |
| Gehrman | 2013 | 0 | 1 | 1 | 1 | 1 | 0 | 1 | 1 | 1 | 7 |
| Maglione | 2014 | 0 | 1 | 1 | 1 | 1 | 0 | 1 | 1 | 1 | 7 |
| Van Mill | 2014 | 1 | 1 | 1 | 1 | 1 | 0 | 1 | 1 | 0 | 7 |
| Fernandez | 2015 | 1 | 1 | 1 | 1 | 1 | 0 | 1 | 1 | 1 | 8 |
| Furihata | 2017 | 1 | 1 | 1 | 1 | 1 | 0 | 1 | 1 | 0 | 7 |
| Jackowska | 2017 | 1 | 1 | 1 | 1 | 1 | 0 | 1 | 1 | 0 | 7 |
| Li | 2017 | 1 | 1 | 1 | 1 | 1 | 0 | 1 | 1 | 1 | 8 |
| Lippman | 2017 | 1 | 1 | 1 | 1 | 1 | 0 | 1 | 1 | 0 | 7 |
| Plante | 2017 | 1 | 1 | 1 | 1 | 1 | 0 | 1 | 1 | 0 | 7 |
| Jing | 2020 | 1 | 1 | 1 | 1 | 1 | 0 | 1 | 1 | 1 | 8 |
